# Author Correction: Characterization of a second class Ie ribonucleotide reductase

**DOI:** 10.1038/s42003-025-07982-4

**Published:** 2025-04-01

**Authors:** Juliane John, Daniel Lundin, Rui M. Branca, Rohit Kumar, Vivek Srinivas, Hugo Lebrette, Martin Högbom

**Affiliations:** 1 Department of Biochemistry and Biophysics, Stockholm University, Arrhenius Laboratories for Natural Sciences, Stockholm, Sweden; 2 Cancer Proteomics Mass Spectrometry, Department of Oncology-Pathology, Science for Life Laboratory, Karolinska Institutet, Solna, Sweden; 3 Laboratoire de Microbiologie et Génétique Moléculaires, Centre de Biologie Intégrative, CNRS – University of Toulouse, Toulouse, France

**Keywords:** Proteins, Structural biology

Correction to: *Communications Biology* 10.1038/s42003-025-07565-3, published online 22 February 2025

In the version of the article initially published, there was an error in the structure presented in Fig. 4 where a C atom was missing in the schematic representation of the nucleotides. Additionally,  in the Acknowledgements, Inna Rozman Grinberg was not thanked for help with activity assays. The figure and Acknowledgements have been updated in the HTML and PDF versions of the article.

