# Supplementary material for: Characterization of a second class Ie ribonucleotide reductase
Source: Commun Biol. 2025 Feb 22;8:281. doi: 10.1038/s42003-025-07565-3 (PMC11846895; doi:10.1038/s42003-025-07565-3)
Supplement: Supplementary file 1 — Supplementary information [file 42003_2025_7565_MOESM1_ESM.pdf]

Supplementary information for:

Characterization of a second class Ie ribonucleotide reductase

Juliane John[1], Daniel Lundin[1], Rui M. Branca[2], Rohit Kumar[1], Vivek Srinivas[1], Hugo Lebrette[3]*, Martin Högbom[1]*

[1]Department of Biochemistry and Biophysics, Stockholm University, Arrhenius Laboratories for Natural Sciences, Stockholm, Sweden.

[2]Cancer Proteomics Mass Spectrometry, Department of Oncology-Pathology, Science for Life Laboratory, Karolinska Institutet, Solna, Sweden.

[3]Laboratoire de Microbiologie et Génétique Moléculaires, Centre de Biologie Intégrative, CNRS – University of Toulouse, Toulouse, France.

*corresponding authors: hugo.lebrette@univ-tlse3.fr, hogbom@dbb.su.se

Supplementary Table 1. Distribution of R2e$_{QSK}$ and R2e$_{VPK}$ genes in GTDB species representative genomes

***nrdF* variant**	**operon**	**nr found**
QSK	EF	5
	EFF	1
	F	28
	FIEF	1
	HIEF	17
	IEEEFF	1
	IEF	106
	IF	104
	IFF	1
	IFIEF	11
VPK	FE	2
	FIE	125
	HFIE	1

Supplementary Table 2. Results of TXRF measurements. Metal content for Mn, Fe, Co Ni, Cu and Zn was measured and the ratio of metal/protein determined

Sample name	protein conc. [µM]	elem.	measured metal conc. [mg/l] 1	2	3	avrg	metal [uM]	σ (metal) [uM]	metal/R2 [%]
R2	333	Mn	0.018	0	0	0.006	0.000	0.109	0.03
		Fe	0.036	0.141	0.032	0.070	0.573	1.248	0.37
		Co	0	0	0	0.000	0.000	0.000	0.00
		Ni	1.993	2.026	1.987	2.002	33.854	34.109	10.25
		Cu	0.157	0.169	0.163	0.163	2.565	2.565	0.77
		Zn	0.09	0.498	0.084	0.224	1.285	3.426	1.03
R2+NrdH	154	Mn	0	0	0	0.000	0.000	0.000	0.00
		Fe	0.025	0.033	0.026	0.028	0.466	0.501	0.33
		Co	0	0	0	0.000	0.000	0.000	0.00
		Ni	0.703	0.68	0.69	0.691	11.756	11.773	7.66
		Cu	0.067	0.066	0.058	0.064	0.913	1.002	0.65
		Zn	0.112	0.105	0.107	0.108	1.637	1.652	1.08
R2+NrdI	265	Mn	0.025	0.01	0.023	0.019	0.419	0.352	0.13
		Fe	0.034	0.037	0.047	0.039	0.842	0.704	0.27
		Co	0	0.006	0	0.002	0.000	0.036	0.01
		Ni	5.5	5.219	5.402	5.374	92.037	91.554	34.52
		Cu	0.281	0.263	0.274	0.273	4.312	4.291	1.62
		Zn	0.259	0.247	0.262	0.256	4.007	3.916	1.48
R2+NrdI+NrdH	301	Mn	0	0.019	0	0.006	0.000	0.115	0.04
		Fe	0.083	0.035	0.039	0.052	0.698	0.937	0.31
		Co	0	0	0	0.000	0.000	0.000	0.00
		Ni	4.787	3.842	3.782	4.137	64.436	70.484	23.40
		Cu	0.445	0.364	0.358	0.389	5.634	6.122	2.03
		Zn	0.706	0.575	0.561	0.614	8.581	9.391	3.12
R2+R1+NrdI+NrdH	574	Mn	0	0	0.013	0.004	0.237	0.079	0.01
		Fe	0.089	0.045	0.046	0.060	0.824	1.074	0.19
		Co	0	0	0	0.000	0.000	0.000	0.00
		Ni	2.514	2.567	2.571	2.551	43.803	43.457	7.58
		Cu	0.362	0.37	0.363	0.365	5.712	5.744	1.00
		Zn	0.61	0.605	0.618	0.611	9.452	9.345	1.63

Supplementary Table 3. Surface areas and salt bridges between homodimers of different R2s (see Material and Methods for details). For most entries a range is given since the calculated area often differs from monomer to monomer. *For PDB entries with one monomer in the ASU the crystallographic symmetry mate was generated and used for the calculation

RNR Class	Organism	PDB ID	surface area Å^2	salt bridges
**Ia**	*Clostridium botulinum*	6ZJK	2755-2796	14-16
	Escherichia coli	1MXR	3020-3046	0
	Homo sapiens	3OLJ	1523-1540	0
	Saccharomyces cerevisiae	1SMQ	1183-1198	0
	Plasmodium vivax	2O1Z	1200-1213	4
	Plasmodium yoelii	2P1I	672-764	0
	Aquifex aeolicus	7AIK*	2882	10
**Ib**	*Bacillus anthracis*	6QO9	2187-2188	0
	Bacillus cereus	4BMU	2186-2228	0
	Corynebacterium ammoniagenes	3MJO	2622-2641	2
	Bacillus subtilis	4DR0	1621-1735	0
	Escherichia coli	3N37*	1816	4
	Salmonella enterica	1R2F	1741-1746	0
	Mycobacterium tuberculosis	1UZR	1978-1990	0
	Streptococcus sanguinis	4N83	2077-2144	1-2
**Ic**	*Saccharopolyspora erythraea*	6Y2N*	2014	6
	Chlamydia trachomatis	1SYY*	2495	6
**Id**	*Flavobacterium johnsoniae*	6CWO	1133-1151	2
	Leeuwenhoekiella blandensis	5OLK	1062-1097	6
**Ie**	*Aerococcus urinae*	6EBP	2623-2633	3
	Mesoplasma florum	6GP2	2633-2656	0
	Gardnerella vaginalis		4224-4348	4-6

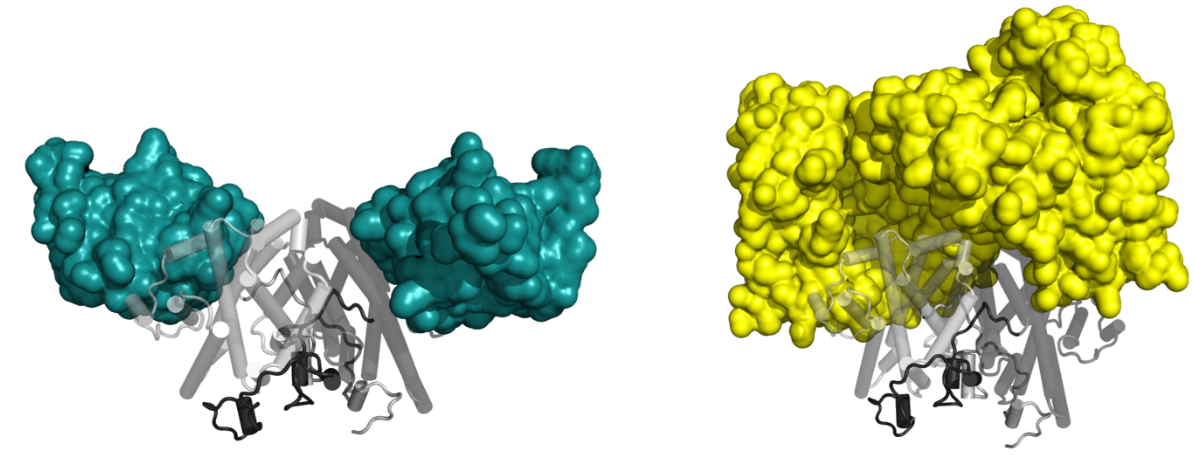

Supplementary Figure 1. **Predicted binding of *Bv*R1 (yellow) and *Bv*NrdI (cyan) to *Bv*R2e (grey)**. The N-termini are on the opposite side of both complexes. They are shown as opaque cartoon and the rest of R2 is transparent for clarity. The figure was generated by aligning the Alphafold prediction for *Bv*R1 (AFDB ID: AF-I4LQF4-F1) to the *E. coli* class Ia R1-R2 complex (PDB ID: 6W4X) and the Alphafold prediction for *Bv*NrdI (AFDB ID: AF-I4LQF3-F1) to *B. cereus* class I b R2-NrdI complex (PDB ID: 7Z3D) with the *Bv*R2.

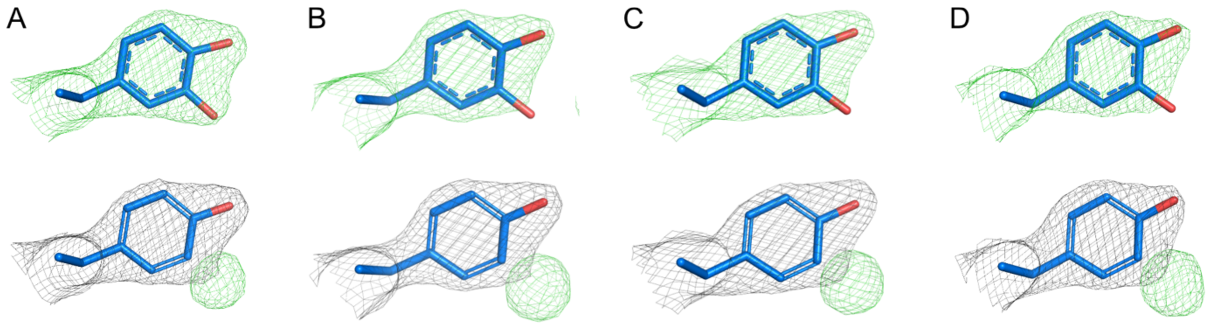

Supplementary Figure 2. **Detailed maps of residue 150 in the *Bv*R2-DOPA structure.** The residues of four different monomers in the asymmetric unit are shown under their chain names. For the top row, a composite omit map was calculated and is contoured at 2 σ, showing residue 150 modelled as DOPA. For the bottom row, residue 150 was modelled as a tyrosine, the 2Fo-Fc map shown in grey is contoured at 2 σ and the Fo-Fc shown in green is contoured at 4 σ.

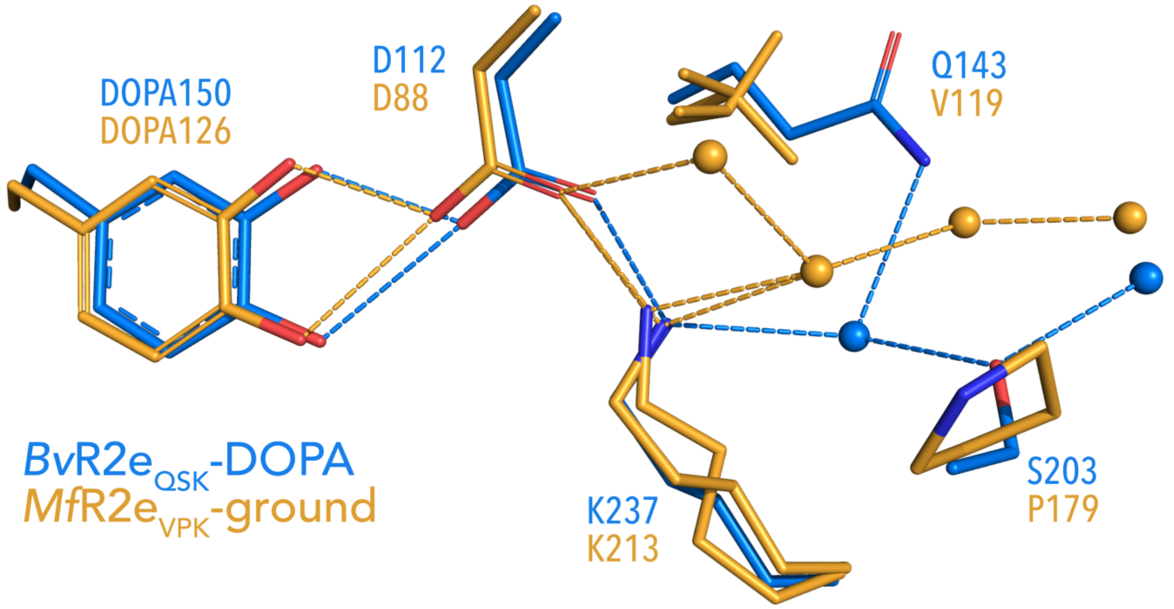

Supplementary Figure 3. **Differences in hydrogen bond network and water structure between $R2e_{QSK}$ and $R2e_{VPK}$.** Overlay of both structures highlights the more extended water network in the VPK version of R2e. Waters are shown as spheres and polar bonds as dashed lines in the colour corresponding to their structure (*Mf*R2e PDB code: 8BT4).
