# Supplementary material for: Characterization of a second class Ie ribonucleotide reductase
Source: Commun Biol. 2025 Feb 22;8:281. doi: 10.1038/s42003-025-07565-3 (PMC11846895; doi:10.1038/s42003-025-07565-3)
Supplement: Supplementary file 2 — Description of Additional Supplementary File [file 42003_2025_7565_MOESM2_ESM.pdf]

Description Of Additional Supplementary File

File name: Supplementary Data 1

Description: Distribution of $R2e_{QSK}$ and $R2e_{VPK}$ genes in GTDB species representative genomes, and source data for all graphs in the main figures.
